# Association between one-carbon metabolism indices and DNA methylation status in maternal and cord blood

**DOI:** 10.1038/s41598-018-35111-1

**Published:** 2018-11-15

**Authors:** Anna K. Knight, Hea Jin Park, Dorothy B. Hausman, Jennifer M. Fleming, Victoria L. Bland, Gisselle Rosa, Elizabeth M. Kennedy, Marie A. Caudill, Olga Malysheva, Gail P. A. Kauwell, Andrew Sokolow, Susan Fisher, Alicia K. Smith, Lynn B. Bailey

**Affiliations:** 1 Genetics and Molecular Biology Program, Emory University, Atlanta, GA USA; 2 Department of Foods and Nutrition, University of Georgia, Athens, GA USA; 3 Department of Environmental Health, Rollins School of Public Health, Emory University, Atlanta, Georgia USA; 4 Division of Nutritional Sciences, Cornell University, Ithaca, NY USA; 5 Food Science and Human Nutrition Department, University of Florida, Gainesville, FL USA; 6 Piedmont Athens Regional Midwifery, Athens, GA USA; 7 Gynecology and Obstetrics, Emory University School of Medicine, Atlanta, GA USA

## Abstract

One-carbon metabolism is essential for multiple cellular processes and can be assessed by the concentration of folate metabolites in the blood. One-carbon metabolites serve as methyl donors that are required for epigenetic regulation. Deficiencies in these metabolites are associated with a variety of poor health outcomes, including adverse pregnancy complications. DNA methylation is known to vary with one-carbon metabolite concentration, and therefore may modulate the risk of adverse pregnancy outcomes. This study addresses changes in one-carbon indices over pregnancy and the relationship between maternal and child DNA methylation and metabolite concentrations by leveraging data from 24 mother-infant dyads. Five of the 13 metabolites measured from maternal blood and methylation levels of 993 CpG sites changed over the course of pregnancy. In dyads, maternal and fetal one-carbon concentrations were highly correlated, both early in pregnancy and at delivery. The 993 CpG sites whose methylation levels changed over pregnancy in maternal blood were also investigated for associations with metabolite concentrations in infant blood at delivery, where five CpG sites were associated with the concentration of at least one metabolite. Identification of CpG sites that change over pregnancy may result in better characterization of genes and pathways involved in maintaining a healthy, term pregnancy.

## Introduction

One carbon metabolism is essential for a variety of cellular processes ranging from DNA replication and methylation to amino acid synthesis, erythropoiesis, and vitamin metabolism^1–3^. One carbon metabolism requires serine, folate, and methionine, making the folate and methionine cycles highly intertwined and vital for cellular processes^3,4^. Both dietary folate and folic acid, the synthetic version used in enriched/fortified foods and supplements, must be reduced to biologically active forms, including 5-methyl-tetrahydrofolate (5meTHF)^1^. Folate is converted to dihydrofolate and then tetrahrydrofolate before becoming biologically active as 5meTHF, which can then donate a methyl group for the remethylation of homocysteine to methionine, bridging the folate and methionine pathways. Methionine is a key amino acid and source of methyl groups as it is converted to *S*-adenosylmethionine (SAM), which becomes *S*-adenosylhomocysteine (SAH) after donating a methyl group^1,4,5^. These one-carbon metabolites can serve as indicators of folate concentrations in blood and play an important role in epigenetic regulation^6^.

Deficiencies in one carbon metabolites have been associated with increased risk for a range of poor health outcomes, including vascular disease, megaloblastic anemia, and certain types of cancer in adults^7^. The consequences of deficiencies in one-carbon metabolism are even more pronounced during intrauterine development, as folate deficiencies have been associated with a range of pregnancy complications including preterm birth and neural tube defects^1,7–11^.

The requirement for folate during pregnancy is substantially greater than folate requirements for non-pregnant women^12^. This increase in demand is likely related to folate requirements for the growth and development of the fetus and placenta, as well as the increased blood volume associated with the pregnant state^1,9^. A recent study of steady-state levels of folic acid demonstrated that pregnancy alters folate pharmacokinetics, suggesting folate kinetics are non-linear and change with gestational age^13^. Other studies have shown both an increase and no change in folate catabolism with pregnancy^12,14–16^, leaving open questions about the influence of pregnancy on one-carbon metabolism.

In addition to the adverse pregnancy outcomes, folate deficiency has also been associated with changes in DNA methylation^8,9,17–20^. One of the most striking studies of DNA methylation and maternal folate concentrations examined fetal tissue from pregnancy terminations due to the presence of neural tube defects and elective terminations as controls^8^. This study found that the distribution of DNA methylation in several tissues, including brain, was different in cases and controls, and that DNA methylation in the brain from cases and controls was correlated with maternal folate concentrations, with maternal folate being lower in blood samples from women carrying fetuses with neural tube defects^8^. However, several studies have failed to find associations between many candidate gene methylation levels and one-carbon metabolite concentrations^21,22^. These studies demonstrate the importance of the interplay between DNA methylation and folate metabolism in potentially modulating risk of adverse pregnancy outcomes.

The current study aimed to address changes in one-carbon metabolism indices and DNA methylation during pregnancy, associations between maternal blood and cord blood metabolites and DNA methylation, and the associations between DNA methylation and metabolite concentration. To address these aims, we measured one-carbon metabolism indices and DNA methylation across the genome during the first trimester and at delivery in pregnant women and in umbilical cord blood of their offspring at delivery.

## Results

Twenty-four pregnant women participated in the study. Participant characteristics are presented in Table 1. All mothers were healthy during pregnancy and all infants were full term with Apgar scores at delivery within the normal range. Compliance with taking their supplements during pregnancy, as measured by counting returned pills, was 92.1%.

**Table 1:** Participants characteristics.

	Range	Mean ± SD
Maternal Age (years)	19–35	26.83 ± 5.34
Pre-pregnancy BMI (kg/m^2^)	18.70–35.70	25.51 ± 4.94
Gestational Weeks at First Visit (weeks)	3.71–11.57	7.45 ± 1.74
Gestational Weeks at Delivery (weeks)	38.10–42.90	40.05 ± 1.21
Maternal *MTHFR* genotype	14 (58%) CC, 5 (21%) CT, 5 (21%) TT	
Infant *MTHFR* genotype*	10 (42%) CC, 10 (42%) CT, 3 (13%) TT	
Race/ethnicity	11 (45.8%) Caucasian, 9 (37.5%) Hispanic	
	2 (8.3%) African American, 2 (8.3%) Other	

*Missing data for one subject.

### Changes of one-carbon metabolism indices over pregnancy

One-carbon metabolite indices have been shown to change over pregnancy^5,13,14,23^. This study examined the changes in one-carbon metabolism indices between the first trimester and delivery in pregnant women who consumed prenatal supplements as recommended by Institute of Medicine (IOM). Of the 13 metabolites measured, only 5 indices (38.4%) showed significant changes over pregnancy, with choline, SAH, and RBC folate increasing during pregnancy and Vitamin B12 and betaine decreasing during pregnancy (Table 2).

**Table 2:** Changes in one-carbon metabolism indices and cellular composition over pregnancy.

Metabolite	T statistic	P-value
5-methyl-tetrahydrofolate (5meTHF)	1.55	0.14
Betaine	−8.22	5.28e-8
Choline	5.41	2.3e-5
dimethylglycine (DMG)	−1.63	0.11
Homocysteine	−1.54	0.13
Methionine	1.38	0.18
red blood cell (RBC) folate	5.12	3.9e-5
S-adenosylhomocysteine (SAH)	2.77	0.011
S-adenosylmethionine (SAM)	2.00	0.058
SAM/SAH	−0.21	0.84
Serum Folate	1.30	0.21
trimethylamine N-oxide (TMAO)	−0.21	0.83
Vitamin B12	−2.90	0.008
**Cell Type**	**T statistic**	**P-value**
B cell	−2.19	0.04
Monocytes	2.03	0.06
Natural Killer	−1.49	0.15
CD8+T	−2.86	0.009
CD4+T	−2.96	0.008

**Figure 1:**
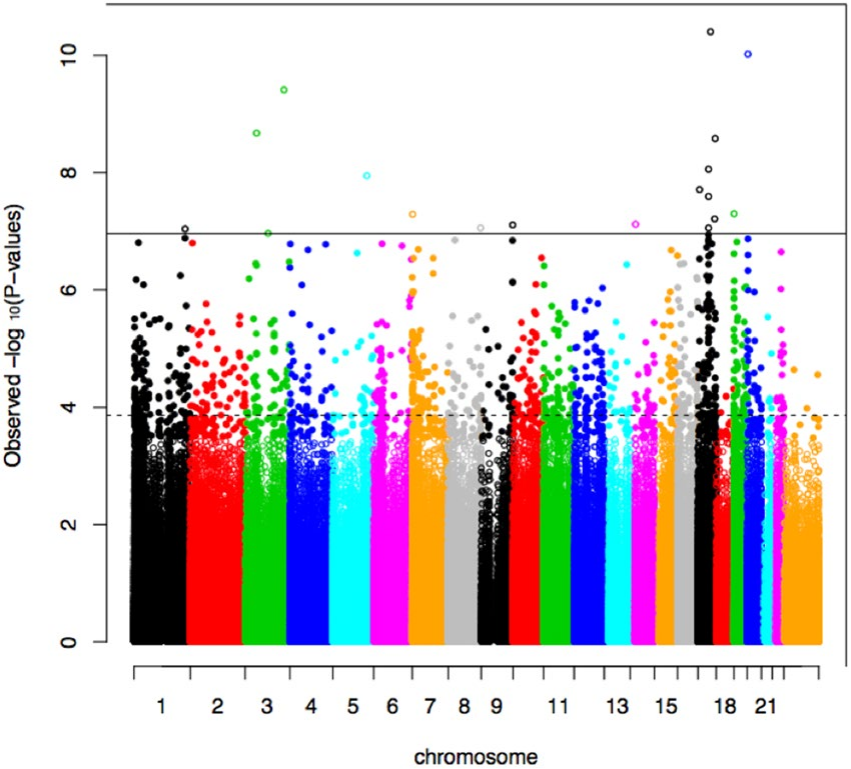
Manhattan plot showing association of CpG sites with gestational weeks at sample collection. Dotted horizontal line indicates experiment-wide significance, based on a false discovery rate (FDR) of 5%. Solid horizontal line indicates Bonferroni significance.

In addition to one-carbon metabolism indices, maternal DNA methylation also changes throughout pregnancy. In this longitudinal analysis, the methylation level of 993 CpG sites changed significantly, with 116 (11.7%) increasing and 877 (89.3%) decreasing over the course of pregnancy (Fig. 1). These CpG sites were not enriched for any particular biological processes, but were associated with molecular functions related to DNA binding and transcription factors (p < 0.05). Additionally, 613 of the 993 CpG sites were associated with gene expression in an independent cohort, which is significantly more than would be expected by chance (p < 2.2 × 10^−16^)^24^.

**Figure 2:**
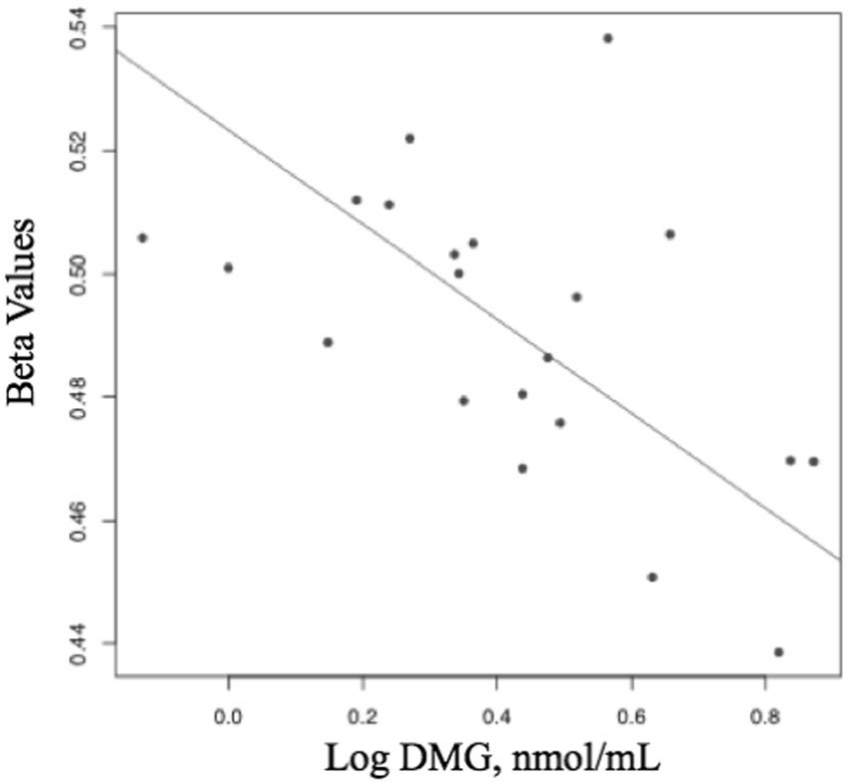
Association between methylation of cg23089643 and log dimethylglycine (DMG) at delivery in pregnant women.

Of the 993 CpG sites whose methylation levels change over pregnancy, only one (cg23089643) was negatively associated with plasma DMG levels in maternal blood at delivery (Fig. 2). This CpG site is not located near a gene, making the clinical significance of this finding uncertain. However, methylation levels at this CpG site was also associated with betaine concentrations in a post-hoc analysis (p < 0.05). No other metabolites were associated with CpG sites that changed in methylation over pregnancy.

### Correlations between maternal and cord blood one-carbon metabolites and DNA methylation

Fetal supply of nutrients is largely dependent on maternal nutrient status^9,25–27^. Accordingly, maternal one-carbon metabolism indices were highly correlated with cord blood indices in this study (p < 0.05, Table 3). Maternal homocysteine, TMAO (trimethylamine Noxide), SAM and 5meTHF concentrations at baseline were significantly associated with cord blood concentrations. Additionally, at delivery, maternal homocysteine, TMAO, 5meTHF, Vitamin B12, choline, methionine and SAH concentrations were associated with cord blood concentrations. These data suggest that maternal one-carbon metabolism indices are reflected in fetal circulation.

**Table 3:** Correlations between cord blood metabolite concentrations and maternal metabolite concentrations at baseline and delivery.

Metabolite	Baseline		Delivery	
	R	P-value	R	P-value
5-methyl-tetrahydrofolate (5meTHF)	0.58	0.0079	0.44	0.04
Betaine	0.11	0.66	0.17	0.75
Choline	0.12	0.62	0.51	0.011
dimethylglycine (DMG)	0.10	0.66	0.73	1.4 × 10^–4^
Homocysteine	0.69	7.8 × 10^−4^	0.69	0.0013
Methionine	−0.09	0.71	0.39	0.077
red blood cell (RBC) folate	0.31	0.28	0.03	0.88
S-adenosylhomocysteine (SAH)	0.4	0.88	0.60	0.003
S-adenosylmethionine (SAM)	0.49	0.027	0.14	0.53
SAM/SAH	0.36	0.12	0.21	0.25
Serum Folate	0.31	0.19	0.41	0.05
trimethylamine N-oxide (TMAO)	0.78	4.48 × 10^−5^	0.74	8.62 × 10^−5^
Vitamin B12	0.35	0.11	0.57	0.00

Maternal DNA methylation at delivery was correlated with cord blood DNA methylation at 28 CpG sites in 19 genes (Table S2). Of note, cord blood methylation was not associated with gestational age at birth, likely due to the limited range of gestational ages represented by this study as shown in Table 1.

### Cord blood metabolite levels associate with DNA methylation

**Figure 3:**
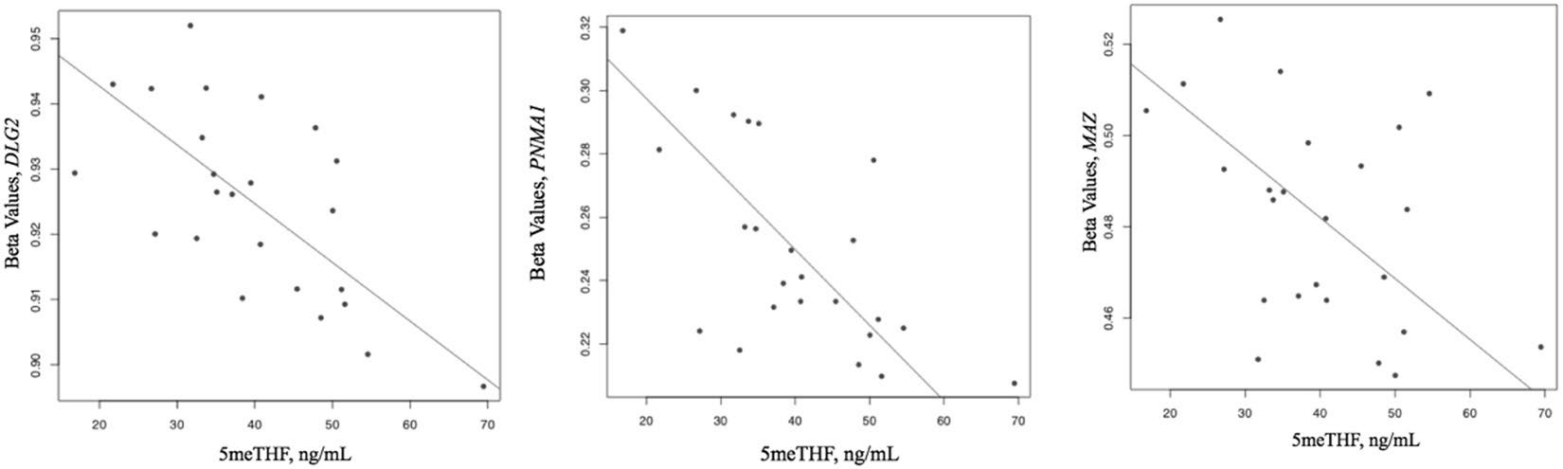
Associations between three CpG sites (cg20694545, cg09238801, cg03527802) and 5-methyl-tetrahydrofolate (5meTHF) in cord blood.

The maternal CpG sites whose methylation levels change over pregnancy are most likely influenced by changing maternal folate and one-carbon metabolite concentrations, making these sites interesting targets to study for associations with cord blood methylation. Therefore, the methylation levels of the 993 CpG sites that change over pregnancy were also tested for associations with cord blood one-carbon metabolism indices. 5meTHF concentrations in cord blood were associated with methylation levels of three CpG sites: cg20694545, cg09238801, cg03527802 (Fig. 3). These CpG sites are located in discs large MAGUL scaffold protein 2 (*DLG2*), paraneonplastic MA antigen 1 (*PNMA1*), and Myc associated zinc finger protein (*MAZ*)^28^, respectively. These genes are involved in neuronal development, inflammation, and hypoxia. We further investigated these CpG sites, as they may be associated with concentrations of other one-carbon metabolites. CpG sites in *DLG2* also associated with methionine, SAH, serum folate, and RBC folate in cord blood and DMG, betaine, and serum folate in maternal blood at delivery (p < 0.05). *MAZ* methylation was also associated with SAM and serum folate (p < 0.05) concentrations in cord blood and with folic acid levels in maternal blood at delivery (p < 0.05). Finally, *PNMA1* methylation was associated with methionine and serum folate in cord blood (p < 0.05).

In cord blood, three CpG sites were associated with DMG: cg13753351, cg00340958, and cg09501509 located in proteasome subunit beta (*PSMB7*), beta-1, 4-galactosyltransferase 5 (*B4GALT5*), and an unannotated region, respectively. These genes have been associated with regulation of the cell cycle and cancer development and progression. These CpG sites were also associated with other metabolites. In cord blood, *B4GALT5* was also associated with the SAM/SAH ratio, *PSMB7* methylation was associated with RBC folate and DMG, and the unannotated site was also associated with DMG. In maternal blood at baseline, there were no additional associations with *B4GALT5* or the unannotated CpG site, but *PSMB7* was also associated with SAM, serum folate, and 5meTHF. In maternal blood at delivery, *B4GALT5* was associated with choline, serum folate, and 5meTHF (p < 0.05), and *PSMB7* methylation was associated with choline, 5meTHF, and TMAO (p < 0.05). The unannotated CpG site was also associated with 5meTHF. Many of these and previously listed associations between metabolites and DNA methylation overlap, suggesting a link between DNA methylation and one-carbon metabolites.

## Discussion

In this study, we show that five out of 13 one-carbon metabolite concentrations studied change over pregnancy. Choline is an essential nutrient required for methylation, synthesis of the neurotransmitter acetylcholine, and cell membrane structure, making it vital for healthy brain development and cognition^29^. The increased choline concentrations during pregnancy observed in this study are consistent with a previous study^25^. Choline is converted into betaine through the actions of choline dehydrogenase and betaine aldehyde dehydrogenase, which has been shown to decrease over pregnancy and turnover rapidly, further confirming our findings^25,26^. Additionally, the increase in RBC folate over pregnancy also confirmed findings of a previous study^27^. To our knowledge, this is the first study examining maternal SAH concentrations over pregnancy, though previous studies have demonstrated higher concentrations of SAH, an inhibitor of methyltransferases, in mothers of fetuses with neural tube defects^30^. Thus, the increase in SAH concentrations with increasing gestation may reflect lower levels of DNA methylation occurrence in late pregnancy compared to earlier pregnancy.

Few studies have examined changes in DNA methylation in maternal blood over pregnancy^31–34^. One recent study identified 45 CpG sites associated with spontaneous preterm birth, which may reflect DNA methylation changes occurring in the weeks between preterm and term birth^34^. However, only two of these CpG sites were both replicated and validated in an independent cohort^34^. Identification of CpG sites whose methylation levels change over pregnancy may result in better characterization of the genes and pathways involved in maintaining a healthy, uncomplicated pregnancy. This may allow for development of DNA methylation-based biomarkers of various physiological changes or complications in pregnancy. In contrast to the above study, we identified 993 CpG sites that change over a wider range of gestational weeks, and were enriched for transcription factors and DNA binding, potentially indicating differential regulation of many genes during pregnancy. These CpG sites may provide insight into how gene regulation changes over pregnancy and how one-carbon metabolites influence regulation of DNA methylation.

The 993 CpG sites that change over pregnancy were interrogated for associations with metabolite concentrations in both maternal and cord blood. One CpG site that was not annotated to a nearby gene was associated with maternal DMG concentrations at delivery. DMG is oxidized in the mitochondria as part of the folate-mediated one-carbon cycle^35^. Though the clinical relevance of this finding is unclear, higher DMG concentrations have previously been associated with low concentrations of maternal folate and may serve as an indicator of maternal folate intake^36^. Three CpG sites, two of which (*PSMB7* and *B4GALT5)* were annotated to nearby genes, were associated with DMG concentrations in cord blood. *PSMB7* has been shown previously to be associated with cell proliferation, the molecular mechanism of bipolar disorder, autophagy, and cancer^37–40^. *B4GALT5* has been associated with cancer progression and drug resistance, especially in gliomas^41–43^. We hypothesized that there would be more associations between DNA methylation and one-carbon metabolite indices. The lack of identified associations could reflect the small sample size of this study or limited power to detect small effect sizes. Additionally, the one-carbon metabolism pathway is very complex and other unmeasured confounding factors may cloud the relationship between DNA methylation and one-carbon metabolism indices in this sample. Therefore, the relationships identified in this study are likely to be very robust and have a relatively large effect size.

In the fetus, low serum 5meTHF, which has been associated with preterm birth, anemia, macrocytosis, and cognitive function^44,45^, was associated with three CpG sites. These CpG sites are located in the *DLG2*, *MAZ*, and *PNMA1*genes. *DLG2* has been associated with Parkinson’s disease, schizophrenia, and other neurological disorders^46,47^. *PNMA1* is expressed in the fetal brain and during neuronal death and has been identified in patients with neurological disorders^48^. *MAZ* has been associated with hypoxia, inflammation, and angiogenesis^49–51^. Thus, these genes are involved in vital developmental processes for fetal growth and development, and associations between 5meTHF and DNA methylation of these genes may reveal a mechanism of how folate supplementation may influence fetal outcome.

The secondary associations interrogated for CpG sites reaching genome-wide significance show that many metabolite-methylation associations show similar patterns. For example, *PSMB7* was associated with 5meTHF in maternal baseline and delivery samples and choline was associated with both *B4GALT5* and *PSMB7* at delivery. These overlapping associations suggest that DNA methylation may be regulated by more than one metabolite level, or that metabolite concentrations may regulate DNA methylation at multiple sites.

Finally, it is interesting to note that maternal and cord blood DNA methylation was correlated for 28 CpG sites. A previous study also found significant associations between maternal and cord blood DNA methylation^32^, potentially providing another mechanism for fetal programming through maternal factors. Maternal and cord blood metabolite concentrations were also correlated for DMG, homocysteine, TMAO, choline, SAH, SAM, Vitamin B12, and 5meTHF, partially confirming results from previous studies^25–27^ and highlighting the importance of maternal metabolite concentrations and placental transfer for cord blood metabolite levels. Cord blood DNA methylation, however, was not associated with gestational age at birth, in contrast to several previous studies^52–54^. This discrepancy is likely due to the small sample size and limited gestational age range at birth in the present study.

Strengths of this study include the use of both maternal blood and cord blood samples, a large number of characterized metabolites, and consistent maternal folate supplement use. However, this study is not without limitations, including having a small sample size and a limited follow-up period.

## Conclusions

Both one-carbon metabolite concentrations and DNA methylation change over pregnancy in maternal blood samples. Maternal blood and cord blood metabolite and DNA methylation levels are also correlated. CpG sites whose methylation levels change over pregnancy were associated with maternal DMG concentrations at delivery and with cord blood 5meTHF concentrations. The genes associated with 5meTHF are likely involved in vital neuronal and developmental processes. However, these associations should be interpreted with caution as we do not have gene expression data to help interpret the functional relevance in changes in DNA methylation. Future studies should examine these associations in larger cohorts and over a wider range of one-carbon metabolite concentrations. This study provides a framework for future studies on associations between maternal blood and cord blood metabolites and DNA methylation.

## Methods

### Participant Recruitment

Participants were recruited by midwives from the Piedmont Athens Regional Midwifery Clinic during their first prenatal visit (<12 weeks gestation). Subjects were included if they were between 18–40 years of age and pregnant with a singleton fetus. Subjects were excluded for prior diagnosis with a chronic condition (e.g., diabetes, anemia, hypertension), smoking, use of prescription medication, and presence of pregnancy complications such as pre-eclampsia and gestational diabetes. Participants were instructed not to take any prenatal supplements other than those provided by the research team for the study. Written informed consent was obtained from all subjects. All methods and procedures were approved by the University of Georgia Institutional Review Board on Human Subjects (STUDY00000506) and the Athens Regional Medical Center Institutional Review Board. This study was registered at ClinicalTrials.gov (NCT02124642). All experiments were performed in accordance with relevant guidelines and regulations.

### Supplementation of vitamins, minerals and docosahexaenoic acid (DHA) recommended during pregnancy

Mothers were provided prenatal supplements including vitamins, minerals and DHA. The daily supplements included Vitamin A (700 μg, 10% as beta-carotene), Vitamin C (75 mg), Vitamin D (25 μg), Vitamin E (7.5 mg), Vitamin K (25 μg), Thiamin (1.2 mg), Riboflavin (1.3 mg), Niacin (16 mg), Vitamin B6 (1.7 mg), Vitamin B12 (6 μg), Biotin (1000 μg), Pantothenic acid (5 mg), Folic acid (400 μg or 800 μg), Calcium (380 mg), Iron (28 mg), Iodine (150 μg), Zinc (8 mg), Selenium (27.5 μg), Copper (0.9 mg), Manganese (1.8 mg), Chromium (25 μg), and DHA (200 mg). Participants were instructed to take the supplements daily at around the same time each day. Supplements were provided at study enrollment and exchanged monthly at each follow-up prenatal visit throughout pregnancy. Compliance was measured by counting returned pills.

Two doses of folic acid were randomly assigned to subjects to investigate the impact of the folic acid dose on the concentrations of one-carbon metabolites. However, no significant differences were observed throughout the pregnancy as well as at delivery between groups. Therefore, the association between blood markers and DNA methylation were analyzed in all subjects regardless of the folic acid doses.

### Demographic and Health Information

Maternal demographic and health information was acquired from medical record abstraction, including age, race, number of previous pregnancies, medical history, prescription and non-prescription drug use, weight during pregnancy, and height. Pre-pregnancy weight was self-reported. BMI was calculated using the “Adult BMI Calculator” from the Centers for Disease Control and Prevention (https://www.cdc.gov/healthyweight/assessing/bmi/adult_bmi/english_bmi_calculator/bmi_calculator.html). Patients also completed a health-behavior questionnaire by phone following enrollment. This questionnaire included information on pre-pregnancy supplementation, consumption of highly fortified foods, smoking and alcohol history, physical activity before pregnancy and amount of time spent outdoors. Neonatal characteristics, including date and mode of delivery, gestational age at birth, gender, anthropometric measurements, and Apgar score, were obtained via medical record abstraction.

### Blood Sample Collection

Non-fasting venous blood was collected at enrollment and delivery. Cord blood was collected from the umbilical vein by trained nurses in the Labor and Delivery Unit. Blood was collected in EDTA-coated tubes and serum separator tubes that were wrapped in foil, stored on ice, and processed within two hours of collection. Serum was allowed to clot for 30 minutes at room temperature and centrifuged for 15 minutes at 1200 × g. One mL of serum was removed and combined with 71.4 μL of 7% ascorbic acid solution, then divided into two 500 μL aliquots for serum folate analysis. A 100 μL sample of whole blood was added to 1.0 mL of 1% ascorbic acid, wrapped in foil to protect from light, mixed on a rotating platform for 30 minutes, and divided into 500 μL aliquots for RBC folate analysis. Ten mg of ascorbic acid was added to 1 mL of serum and subsequently divided into two 500 μL aliquots for folic acid analysis. Samples for analysis of RBC folate, serum folate, folic acid, and other one-carbon metabolites were stored at −80 °C prior to shipping on dry ice to analytical labs. Complete blood count (CBC) samples were analyzed on fresh whole blood samples by LabCorp (Birmingham, AL).

### Blood sample analysis

Serum and red blood cell folate concentrations were determined by microbiological assay using *Lactobacillus rhamnosus*^55,56^. The inter- and intra-assay coefficients of variation were 7.7% and 6.7%, respectively, for serum folate and 5.1% and 3.3%, respectively for RBC folate. Plasma choline, betaine, dimethylglycine (DMG), and trimethylamine *N-*oxide (TMAO) concentrations were determined using LC-MS/MS stable isotope dilution methods as described by Yan *et al*.^57^ using a TSQ Quantum mass spectrometer (Thermo) with refrigerated Accela autosampler (Thermo) and Accela pump with degrasser (Thermo). *MTHFR 677* *C* → *T* (rs18001133) genotype was determined after purifying PCR products (QUIquick PCR Purification kit) and sequencing DNA templates with an Applied Biosystems Automated 3730 DNA analyzer (Applied Biosystems) by the Georgia Genomics Facility (Athens, GA).

### DNA methylation

DNA methylation was interrogated for each site on the HumanMethylation450 BeadChip. Briefly, 1 μg of DNA was processed and hybridized to the HumanMethylation450 BeadChip (Illumina, San Diego, CA) according to manufacturer’s instructions. Quality control was performed using the R package CpGassoc^58^. CpG sites with low signal or missing data for more than 10% of samples were removed, and any sample with missing data for greater than 5% CpG sites was removed. Cross-reactive probes were removed^59^. One sample failed quality control. After quality control, 453,955 CpG sites were included in subsequent analyses. Beta values (β) were calculated for each CpG site as the ratio of methylated (M) to methylated and unmethylated (U) signal: β = M/M + U. Beta-mixture quantile normalization was performed as previously described^60^. The R package ComBAT was used to adjust β values for Chip and Array position^61^. Cell type proportions were estimated from DNA methylation data using the R package minfi^62^. A logit transform was then performed on the β values for the epigenome-wide analyses. DNA methylation data can be accessed through NCBI’s Gene Expression Omnibus, GSE80310 for cord blood and GSE114935 for maternal blood.

### Statistical Analysis

Metabolite concentrations were logarithmically transformed if they did not show a normal distribution (betaine, choline, SAH, DMG, homocysteine, methionine, folic acid, TMAO). Changes in metabolite concentrations over pregnancy were assessed using linear mixed effects models for each metabolite and gestational weeks of sample collection, controlling for repeated measures on the same subject.

The R package CpGassoc was used to perform an epigenome-wide association study (EWAS) for the association of DNA methylation at each CpG site on the array with gestational weeks at sample collection^58^. This analysis was performed using a linear mixed-effects model controlling for maternal age, cell type proportion (CD8T+, CD4T+, monocytes, natural killer, B cells), and repeated measures on the same subject. The false discovery rate was controlled at 5% using q values. Pathway analysis was performed using DAVID^63^. A Benjamini-adjusted p-value was used to account for multiple tests.

The CpG sites reaching genome-wide significance in the EWAS were carried on to further analyses of DNA methylation and metabolite concentrations. Linear regressions were used to assess associations of each significant CpG site and each metabolite level in maternal baseline, maternal delivery, and cord blood samples separately. The linear regression controlled for cell type and age in maternal analyses and cell type and sex in analyses of cord blood.

Pearson’s correlation was calculated between maternal baseline and cord blood samples and maternal delivery and cord blood samples. The association between maternal methylation at delivery and cord blood methylation was assessed using linear regression for each CpG site, controlling for cell type and neonatal sex. The false discovery rate was controlled at 5%.

## Electronic supplementary material

Supplementary Tables
