# Supplementary material for: Association between one-carbon metabolism indices and DNA methylation status in maternal and cord blood
Source: Sci Rep. 2018 Nov 15;8:16873. doi: 10.1038/s41598-018-35111-1 (PMC6237996; doi:10.1038/s41598-018-35111-1)
Supplement: Supplementary file 1 — Supplementary Tables [file 41598_2018_35111_MOESM1_ESM.pdf]

Association between one-carbon metabolism indices and DNA methylation status in maternal and cord blood

Anna K. Knight[1*], Hea Jin Park[2*], Dorothy B. Hausman[2], Jennifer M. Fleming[2], Victoria L. Bland[2], Gisselle Rosa[2], Elizabeth M. Kennedy[3], Marie A. Caudill[4], Olga Malysheva[4], Gail PA. Kauwell[5], Andrew Sokolow[5], Susan Fisher[6], Alicia K. Smith[1,7], Lynn B. Bailey[2]

[1]Genetics and Molecular Biology Program, Emory University, Atlanta, GA, USA
[2]Department of Foods and Nutrition, University of Georgia, Athens, GA, USA
[3]Department of Environmental Health, Rollins School of Public Health, Emory University, Atlanta, Georgia, USA
[4]Division of Nutritional Sciences, Cornell University, Ithaca, NY, USA
[5]Food Science and Human Nutrition Department, University of Florida, Gainesville, FL, USA
Piedmont Athens Regional Midwifery, Athens, GA, USA
[7]Gynecology and Obstetrics, Emory University School of Medicine, Atlanta, GA, USA

* Co-first authors; these authors contributed equally.

Corresponding author:
Alicia K. Smith, PhD
Associate Professor
Gynecology and Obstetrics
Emory University SOM
101 Woodruff Circle NE; Ste 4217
Atlanta, GA 30322
Phone: 404-778-2005
Fax: 404-727-3233
alicia.smith@emory.edu

Supplemental Table 1: Metabolite concentrations at each sampling point.

	Baseline			40 weeks			Cord blood		
**Metabolite**	**Range**	**Mean ± SD**	**N**	**Range**	**Mean ± SD**	**N**	**Range**	**Mean ± SD**	**N**
**5meTHF**	7.29-47.21	25.06±12.83	24	10.91-49.59	30.86 ±10.57	24	16.83-69.48	39.14±11.98	24
**Betaine**	12.06-33.26	20.99±5.80	24	6.34-16.24	11.18 ±2.53	22	15.26-37.06	22.71±5.35	22
**Choline**	3.85-9.46	6.31±1.54	24	6.34-11.79	8.44 ±1.35	22	17.49-69.83	32.58±14.10	22
**DMG**	.95-5.17	2.05±1.16	24	.88-2.39	1.57 ± .40	22	1.49-20.50	3.11±4.13	22
**Homocysteine**	3.19-9.50	5.55±1.72	24	3.10-8.82	4.91 ± 1.74	22	2.65-6.55	4.30±1.22	22
**Methionine**	16.30-40.21	22.58±6.39	24	15.80-37.52	24.47 ±5.66	22	31.03-55.76	41.06±6.16	22
**RBCFolate**	210.3-1860.0	1146±480.07	24	1336-5095	2216 ±877.56	23	755-2765	1852±609.45	23
**SAH**	2.85-9.79	5.98±2.06	24	1.48-14.37	8.58 ±3.26	24	11.25-53.32	24.55±10.93	22
**SAM**	32.100-147.50	66.26±28.73	24	38.62-152.30	82.76 ±28.56	24	106.2-286.8	187.3±51.60	22
**SAM/SAH**	5.984-47.21	25.06±3.65	24	3.10-27.86	11.06 ±5.40	24	3.69-17.08	8.77±3.64	22
**SerumFolate**	15.70-122.50	59.47±32.25	24	24.20-110.40	71.10 ±23.60	24	36-139.30	86.54±26.33	23
**TMOA**	.53-94.84	5.61±19.45	24	.51-33.66	3.04 ±6.88	22	.64-31.10	3.13±6.65	22
**Vitamin B12**	156.3-792.9	435.3±144.34	24	119.5-594.6	313.8 ±132.97	24	154.6-1682	689.2±320.96	23

Supplemental Table 2: Correlated CpG sites between maternal methylation at delivery and cord blood methylation.

CpG Site	Gene	Chromosome	T value	R2	P-val	FDR
cg16970850	*PINX1*	8	13.41	0.93	9.35E-10	4.24E-04
cg13453244		8	10.75	0.90	1.92E-08	2.90E-03
cg20425384		12	10.87	0.91	1.65E-08	2.90E-03
cg19252607	*BRD1*	22	10.40	0.93	2.98E-08	3.38E-03
cg02909570		16	10.16	0.89	4.04E-08	3.56E-03
cg19322825		7	10.04	0.88	4.71E-08	3.56E-03
cg10410513	*ARHGEF10*	8	9.64	0.88	8.08E-08	5.24E-03
cg09333221		7	9.44	0.89	1.06E-07	6.04E-03
cg01733928		14	9.29	0.88	1.30E-07	6.04E-03
cg22561794	*BTNL8*	5	9.28	0.88	1.33E-07	6.04E-03
cg14240634	*FBRSL1*	12	9.17	0.89	1.53E-07	6.33E-03
cg24014990	*VASH1*	14	9.02	0.88	1.90E-07	6.81E-03
cg27454698		12	9.00	0.89	1.95E-07	6.81E-03
cg02074316		15	8.64	0.85	3.28E-07	9.91E-03
cg05638648	*COL18A1*	21	9.50	0.90	3.26E-07	9.91E-03
cg02715072	*TNXB*	6	8.52	0.86	3.94E-07	1.12E-02
cg16283179	*C19orf29*	19	8.06	0.84	7.88E-07	2.09E-02
cg17766828	*C10orf122*	10	-8.03	0.85	8.28E-07	2.09E-02
cg01012936	*NUAK2*	1	7.89	0.86	1.03E-06	2.33E-02
cg19953917	*SCHIP1*	3	7.92	0.84	9.77E-07	2.33E-02
cg02143936	*MICAL2*	11	7.62	0.85	1.56E-06	3.09E-02
cg13796831	*BAT2L2*	1	-7.64	0.81	1.52E-06	3.09E-02
cg19415746	*NRAP*	10	7.67	0.81	1.45E-06	3.09E-02
cg20896439	*GALNTL1*	14	7.51	0.85	1.85E-06	3.50E-02
cg05368730		12	7.45	0.84	2.03E-06	3.55E-02
ch.22.439136F	*C22orf30*	22	7.47	0.84	1.98E-06	3.55E-02
cg00956252	*CLEC16A*	16	7.34	0.89	2.44E-06	4.09E-02
cg01248855	*C17orf42*	17	7.24	0.81	2.90E-06	4.69E-02
